# Dynamic changes of scleral spur length in different accommodation stimuli states

**DOI:** 10.1038/s41598-021-97754-x

**Published:** 2021-09-13

**Authors:** Liugui Chen, Wei Jin, Xinlei Hao, Xuejie Li, Yiqiao Xing

**Affiliations:** Eye Center, Renmin Hospital of Wuhan University, Jiefang Road 238#, Wuchang District, Wuhan, 430060 Hubei Province China

**Keywords:** Glaucoma, Risk factors

## Abstract

This study aimed to evaluate the scleral spur length (SSL) in response to different accommodation stimuli states, as well as the correlation with Schlemm’s canal (SC) and trabecular meshwork (TM). 74 children were recruited for this study. The 0D, − 4D, and − 8.0 D accommodation stimuli state was achieved by looking at a variable distance optotype. The ciliary muscle (CM), scleral spur (SS), SC, and TM were imaged by swept-source optical coherence tomography. The SSL (Method III) increased significantly from 221.56 ± 30.74 μm at base state to 234.99 ± 30.11 μm at − 4D accommodation stimuli state (*p* = 0.028) and increased to 250.09 ± 29.87 μm at − 8D accommodation stimuli state (*p* = 0.011). Method III had the largest areas under receiver operating characteristic (ROC) curves (0.798, 95% CI 0.721–0.875). Moreover, CM 1, SC, and trabecular meshwork length (TML) were significantly correlated with SSL (Method III) (*p* < 0.05). These findings suggest that the contractile ability and compliance of the SS play an important role in maintaining the morphology of the SC. Moreover, the force of accommodation regulates the SC size by increasing the length of SS.

## Introduction

Primary open-angle glaucoma (POAG) is a common form of glaucoma and one of the most common causes of irreversible blindness in the world^1^. Elevated intraocular pressure (IOP) is a primary risk factor for POAG and is caused by increased aqueous humor outflow resistance somewhere in the trabecular meshwork (TM) and outflow pathway of Schlemm’s canal (SC)^2–4^. Many previous studies have shown that the principal sites of outflow resistance are located in the inner wall of the endothelium of SC, the basement membrane of SC, the underlying extracellular matrix of juxtacanalicular tissue, and the TM^5–7^. Thus, in the clinic, the SC and TM have been considered the most important therapeutic targets for the treatment of POAG.

The scleral spur (SS) has been considered to play an important role in maintaining the structure of the SC and TM, which are key components in the pathways of aqueous humor flow. The SS is a projection of the sclera that is located in the ciliary muscle fibers and at the base of the TM. It has a rigid nature because it contains elastic and collagenous fibers as well as myofibroblast cells^8,9^. Swain DL et al. provided different methods for measuring the scleral spur length (SSL) in pathological sections; they found that compared to healthy subjects, in POAG subjects, the SSL was significantly shorter, suggesting that a shorter SS may be a risk factor for the progression of POAG because short SS would be insufficient for maintaining the structure of the SC and TM^10^. In real-time and in vivo, Li M et al. also found the SSL was significantly shorter in POAG eyes compared to healthy eyes by swept-source optical coherence tomography (SS-OCT). Moreover, the SC area (SCA) was significantly associated with the SSL in both POAG and healthy groups. They suggested that the SS plays an important role in the maintenance of SCA and the SSL could be a novel biomarker for POAG evaluation clinically^11^.

Accommodation is the ability of the visual system to alter its optical power to see objects clearly at a finite distance from the eye, it is done by contraction of the ciliary muscles that are innervated by the parasympathetic nervous system^12^. Previous studies have reported that accommodation stimulation could promote aqueous humour outflow and decreases IOP by expanding the SC size, but the mechanisms underlying these changes are unclear^13,14^. Most researchers suggest that the mechanical effects of ciliary muscles under accommodation states mediate structural changes of TM and SC through the SS. But, changes in SS morphology with accommodation stimuli have not yet been reported in humans. Besides that, pilocarpine can also promote aqueous humor outflow by expanding TM and the inner wall of SC, thus keeping SC lumen open^15,16^. However, the drug’s effectiveness is lost upon severing the anterior attachment of the ciliary muscle from the SS^17^. These results suggested that the SS is an indispensable part of regulating the SC and TM under accommodation states. Moreover, the accommodative ability is at its peak in childhood and gradually declines with age^18,19^. Therefore, in this study, we aimed to evaluate the SSL in children in response to different accommodation stimuli states, as well as the correlation with SC and TM, in an exploration of the mechanisms underlying the regulation of SC.

## Methods

The research was approved by the ethics committee (Renmin Hospital of Wuhan University). The study protocol was registered with chictr.org.cn (ChiCTR-ROC-1900026923). Written informed consent was obtained from the children’s parents. The study followed the tenets of the Declaration of Helsinki. In total, 74 children were recruited from Renmin Hospital of Wuhan University, Hubei Province, China, from June 2020 to September 2020. The inclusion criteria were as follows: (1) children aged from 7 to 14 years old; (2) − 6 D ≥ Drefractive error (RE) ≥  − 0.5 D; (3) best-corrected visual acuity (BCVA) equal to or better than 20/20 (0.00 logMAR, metric Snellen 6/6); and (4) intraocular pressure (IOP) < 21 mmHg. The exclusion criteria were as follows: (1) a history of intraocular disease or systemic disease; (2) a history of ocular or refractive surgery; and (3) amplitude of accommodation (AOA) < 8D.

**Figure 1:**
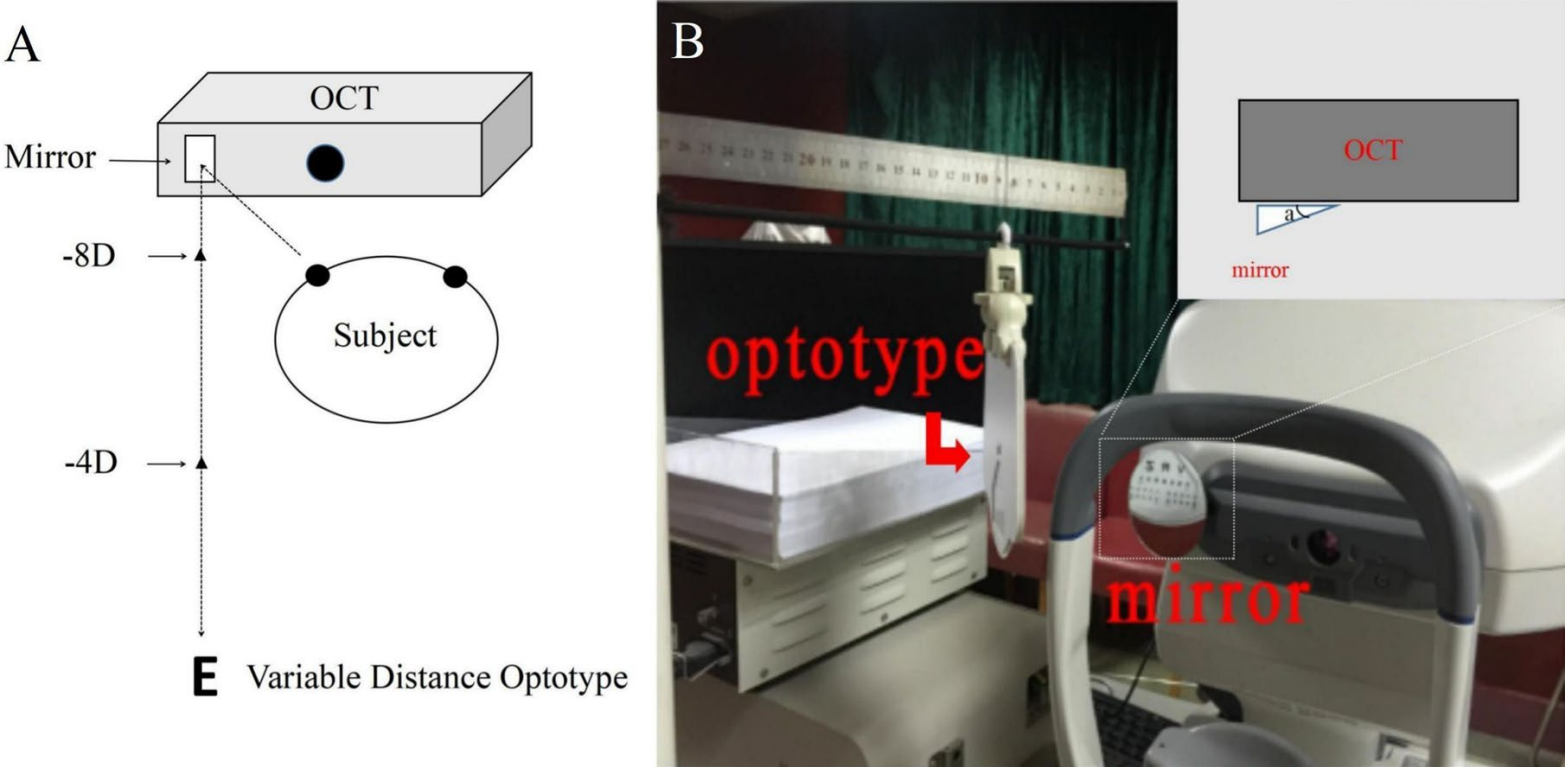
(**A**) Simulated diagram of the setup used to stimulate different accommodation during optical coherence tomography (OCT) imaging. A tilted mirror with a frame carrying a rotation axis was fixed on the OCT machine and used to place the stimuli at different vergences. The participants were instructed to stare at the optotypes through the mirror with their left eye, and right eye was covered with a gauze. Then, the left eye was imaged by OCT. (**B**) According to the individual interpupillary distance and testing items adjusted the tilting angle a of the mirror.

**Figure 2:**
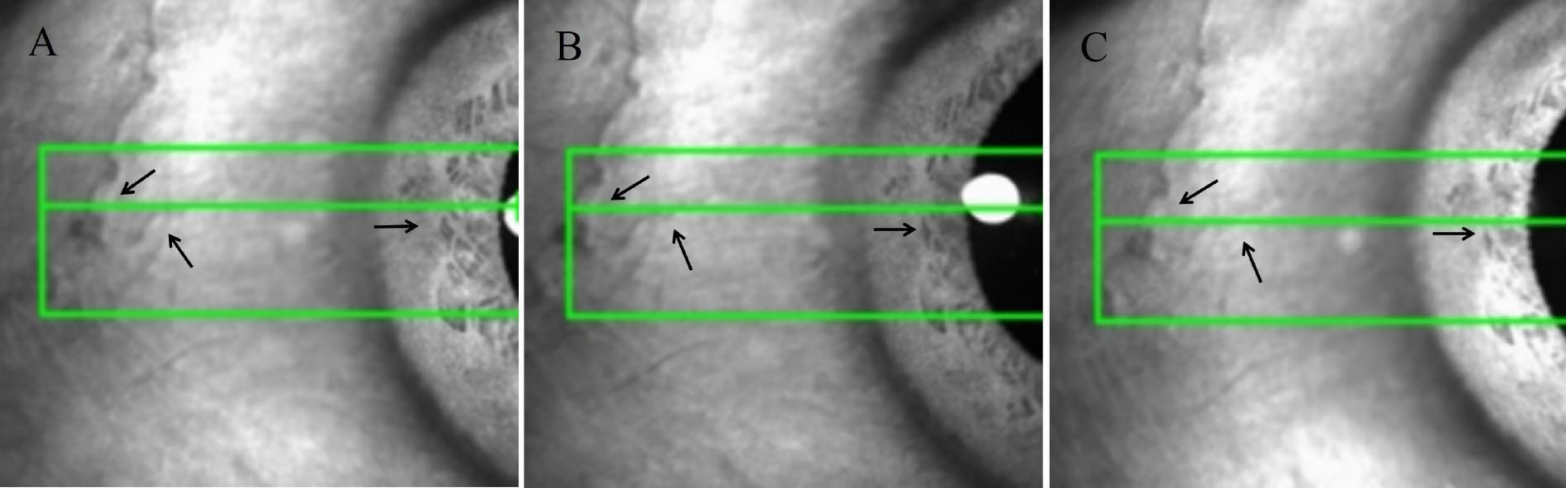
Conjunctival vessels and iris features (black arrow line) were used as landmarks to scan the same cross-section under different states. (**A**) base state. (**B**) -4D accommodation stimuli state. C: -8D accommodation stimuli state.

All participants underwent a serial ophthalmologic examination, including slit-lamp biomicroscopy (Haag-streit, Bern, Swiss), fundus examination, IOP (NIDEK noncontact tonometer RT-2100), subjective refraction, amplitude of accommodation and spherical equivalent refraction (SER, spherical error plus one-half of the cylindrical error) (RT-2100, NIDEK CO. LTD, Gamagori, Japan). The SS-OCT scan used a 1310-nm wavelength with a scan speed of 30, 000 A-scans/s and an axial resolution of less than 10 μm. Participants were imaged with the 3D-angle high-definition scans (dimension, a raster of 64 B-scans each with 512 A-scans over 8 mm). All participants’ left eyes underwent examinations with swept-source OCT (CASIA SS-1000; Tomey Corporation, Nagoya, Japan) in different stimuli states (0D, -4D, -8D). A tilted mirror with a frame carrying a rotation axis was fixed on the external fixation lights. According to the individual interpupillary distance and testing items adjusted the tilting angle of the mirror. The participants were instructed to stare at the optotypes through the mirror with their left eye under the 0D, -4D and the − 8.0 D accommodation (distance based on the formula: 100/ − (− M + X) cm, where X was the refractive error in diopters, M is the accommodative response)^20^ stimulation state, and right eye was covered with a gauze. The optotypes were aligned for a fixed line, to avoid a shift in gaze when moving the optotypes. According to the recommendations of Gabriel, the mean of each 5 s was determined for both accommodative response and pupil size^21^. Thus, the participants must accurately identify the optotypes through the mirror with their left eye after 5 s for each accommodation, then the left eye was scanned three times by the SS-OCT, the measurement took approximately 30 s to complete (Fig. 1). Once the optotypes moves toward or away from the eye, the angular size in retina would be different, this may affect the accommodation response. According to the previous report of Park H et al., the accommodative response to monocular stimulus was 84.6% of the actual refractive stimulus^22^. Therefore, this experiment only gave the same stimulus and optotypes, rather than the same accommodation response. Conjunctival vessels and iris features were used as landmarks to scan the same cross-section under different states to ensure accurate measurements of the CM, SC, TM, and SS (Fig. 2). All the eyes were imaged at nasal positions. The scans of each cross-section were repeated three times, and the best quality image was chosen for analysis. All images were obtained under darkroom conditions (about 40 lx) by the same examiner.

**Figure 3:**
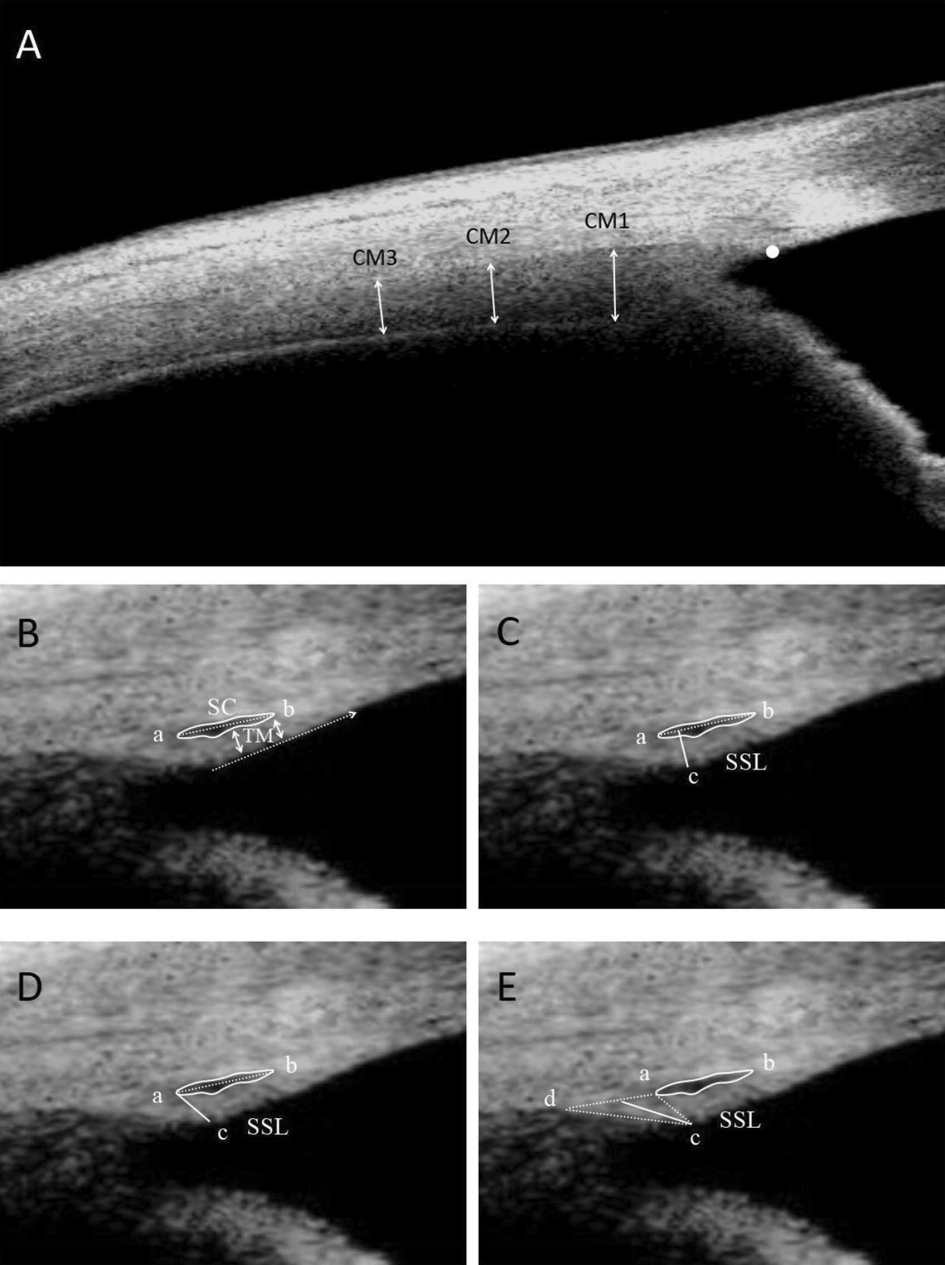
Measured items in the swept- source optical coherence tomography image. (**A**) Thickness of the ciliary muscle width at 1, 2, and 3 mm at posterior to the scleral spur was assessed (CM-1, CM-2, CM-3). Ciliary muscle thickness measurement was made perpendicular to a boundary of the ciliary muscle. (**B**) Schlemm’s canal area (SCA, white loop), Schlemm’s canal length (SCL, a-b dotted line), trabecular meshwork width (TMW, solid arrow), and trabecular meshwork length (TML, dotted arrow line). (**C**–**E**) The scleral spur length (SSL, the solid line) was measured by three different methods.

Thickness of the ciliary muscle width at 1, 2, and 3 mm at posterior to the scleral spur was assessed (CM-1, CM-2, CM-3) (Fig. 3A). Schlemm’s canal area (SCA) was drawn and defined as the black area surrounded by the white outline^11^. Schlemm’s canal length (SCL) was measured from the posterior to anterior SC end point by the a-b dotted line^20^. Trabecular meshwork length (TML) was defined as the dotted arrow line from the tip of the SS to Schwalbe’s line^23^. Trabecular meshwork width (TMW) was defined as the average of the solid arrow line measurements obtained from the anterior and middle points of the SC^23^ (Fig. 3B). The SSL was measured by three different methods from previous studies. Method I^24^ (Fig. 3C): the measurement of the SSL (the white solid line) was drawn perpendicular to the tip of the SS to the a-b dotted line, which connected the anterior and posterior points of the SC. Method II^25^ (Fig. 3D): the SSL (the a-c solid line) was measured from the tip of the SS to the posterior endpoint of the SC along the anterior side of the SS. Method III^11^ (Fig. 3E): the SSL (the white solid line) was drawn from the tip of the SS to the middle of the a-d dotted line, which connected the anterior and posterior points where the sclera curved to form the spur.

20 images from 20 eyes in the participants were randomly chosen. To measure the intraobserver repeatability, a single masked observer (HX) measured the images at two different times with an interval of 3 days, and agreement between the two observations was analyzed. To measure the interobserver reproducibility, the same images were independently evaluated by two observers (HX, LX) blinded to the treatments, and the agreement between them was determined. Intraclass correlation coefficients were calculated using a two-way mixed-effects model. The measurements were performed using ImageJ software (http://imagej.nih.gov/ij/; provided in the public domain by the National Institutes of Health, Bethesda, MD, USA).

All analyses were performed using SPSS statistical software (version 20.0; SPSS, Inc., Chicago, IL, USA). Data are presented as the mean ± standard deviation. Repeated measures analysis of variance (rANOVA) was used to compare variations in the thickness of the ciliary muscle (CM1, CM2, CM3), scleral spur length (SSL), Schlemm’s canal (SCA, SCL), and the trabecular meshwork (TML, TMW) at different accommodation stimuli states (base state, -4D accommodation stimuli state, and -8D accommodation stimuli state). Sphericity was tested with Mauchly's test, and if not statistically significant, Greenhouse–Geisser corrected p-values for F-tests were reported. After a significant F-test, post hoc analysis with Bonferroni correction was used for comparisons between groups. Statistical significance was defined as *p* < 0.05.

### Ethics declarations

The research was approved by the ethics committee (Renmin Hospital of Wuhan University). The study protocol was registered with chictr.org.cn (ChiCTR-ROC-1900026923). Written informed consent was obtained from the children’s parents.

## Results

We computed the sample size needed for rANOVA. A medial level of partial eta square of 0.07 was adopted, which yielded an effect size of about 0.25. A sample size of at least 58 participants was deemed sufficient for a power of 0.80 with 95% confidence. The final sample size was adjusted to 74 based on the 20% participant loss. 7 children were excluded because of low quality images caused by poor patient cooperation. Thus, 67 children (34 male; 33 female) were eventually included in the analyses. The mean values for various variables were as follows: age, 10.91 ± 1.99 years; BCVA, 1.07 ± 0.97 (Snellen chart, Decimal visual acuity); SER, − 2.86 ± 0.89 diopters; IOP, 14.40 ± 2.02 mmHg; and amplitude of accommodation (AOA), 8.86 ± 0.89 diopters (Table 1). The measurements of the SS, SC, and TM parameters showed excellent intraobserver repeatability (ICC range from 0.833 to 0.972) and interobserver reproducibility (ICC range from 0.822 to 0.990) (Table 2).

**Table 1:** Baseline characteristics.

Characteristics	Values
Age (year)	10.91 ± 1.99
**Sex (number of individuals studied)**	
Male	34
Female	33
Best-corrected visual acuity (BCVA, Snellen chart, Decimal visual acuity)	1.07 ± 0.97
Spherical equivalent refraction (SER, diopter)	− 2.86 ± 0.89
Intraocular pressure (IOP, mmHg)	14.40 ± 2.02
Amplitude of accommodation (AOA, diopter)	8.86 ± 0.89

**Table 2:** Interobserver and intraobserver reproducibility of SC, SSL, TM and SC parameter measurements.

Parameter measurements	Interobserver				Intraobserver			
	ICC	Difference	95% CI		ICC	Difference	95% CI	
			Lower	Upper			Lower	Upper
SCA	0.948	33.05	0.874	0.979	0.935	28.19	0.844	0.974
SCL	0.953	2.33	0.886	0.981	0.941	3.48	0.858	0.988
SSL(method I)	0.895	0.67	0.754	0.957	0.833	0.05	0.625	0.930
SSL(method II)	0.859	2.38	0.678	0.942	0.871	2.62	0.703	0.947
SSL(method III)	0.900	3.95	0.766	0.959	0.865	2.52	0.690	0.944
TMW	0.894	0.81	0.752	0.957	0.900	2.76	0.766	0.959
TML	0.822	2.38	0.604	0.925	0.888	3.47	0.738	0.954
CM1	0.990	0.68	0.976	0.996	0.971	0.86	0.930	0.988
CM2	0.980	1.05	0.951	0.992	0.972	2.62	0.931	0.988
CM3	0.955	3.71	0.893	0.982	0.943	1.52	0.866	0.877

Schlemm’s canal area (SCA, μm^2^), Schlemm’s canal length (SCL, μm), Scleral spur length (SSL, μm), Trabecular meshwork width (TMW, μm), Trabecular meshwork length (TML, μm), Ciliary muscle thickness (CM, μm), Intraclass correlation coefficient (ICC), Confidence interval (CI).

**Figure 4:**
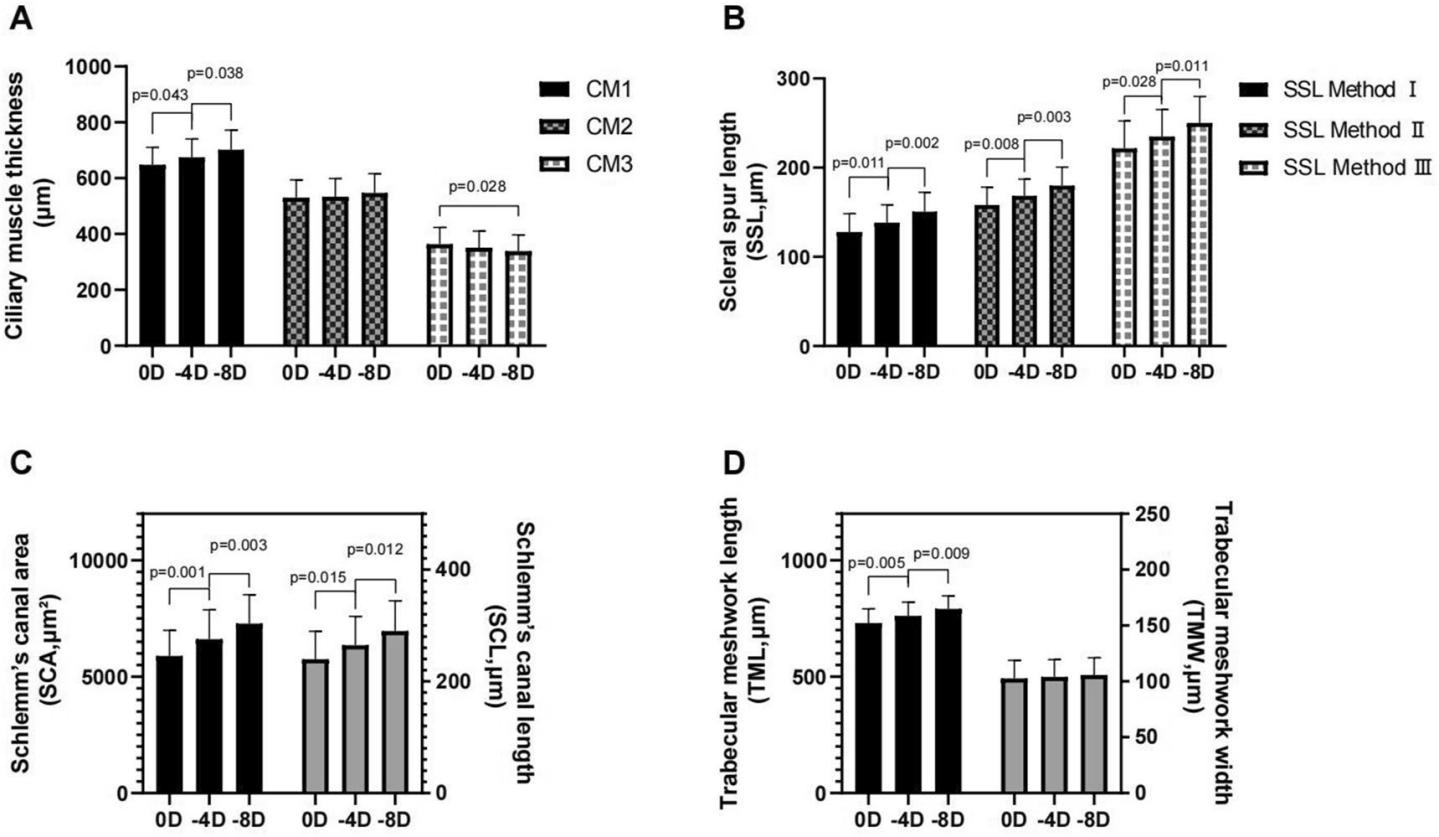
(**A**) CM1 increased significantly with accommodation state. CM2 did not change significantly. Compared to the base state, CM3 decreased at -8D accommodation state. (**B**) SSL that was measured by three different methods increased with accommodation stimulation. (**C**) SCA and SCL increased significantly with accommodation stimulation. (**D**) TML increased significantly with accommodation stimulation, but TMW did not.

The CM1 increased significantly from 648.24 ± 61.85 μm at base state to 674.82 ± 65.98 μm at − 4D accommodation state and increased to 701.92 ± 70.09 μm at − 8D accommodation state. However, compared to 363.85 ± 59.70 μm at the base state, CM3 decreased to 338.47 ± 58.73 μm at − 8D accommodation state. Besides that, CM2 did not change significantly (Fig. 4A).

As Fig. 4B summarizes, the SSL that was measured by three different methods increased with accommodation stimulation. Method I was 127.97 ± 20.32 μm, 138.29 ± 20.11 μm, and 150.73 ± 21.75 μm at 0D, -4D and -8D, respectively. Method II was 158.15 ± 19.97 μm, 168.47 ± 18.72 μm, and 179.79 ± 20.83 μm at 0D, -4D and -8D, respectively. Method III was 221.56 ± 30.74 μm, 234.99 ± 30.11 μm, and 250.09 ± 29.87 μm at 0D, − 4D and − 8D, respectively.

The SCA was 5891.23 ± 1102.66 μm^2^ at the base state, which increased to 6618.19 ± 1255.60 μm^2^ at the − 4.0D accommodation state and increased to 7287.22 ± 1238.50 μm^2^ at the − 8.0D accommodation state. The SCL increased from 239.87 ± 50.02 μm at base state to 264.74 ± 51.77 μm at − 4D accommodation state and increased to 290.15 ± 54.35 μm at − 8D accommodation state. (Fig. 4C). The TML increased with accommodation state (base 730.01 ± 61.80 μm; − 4D 761.75 ± 58.35 μm; − 8D 791.42 ± 55.91 μm). But, TMW did not change significantly (Fig. 4D).

**Figure 5:**
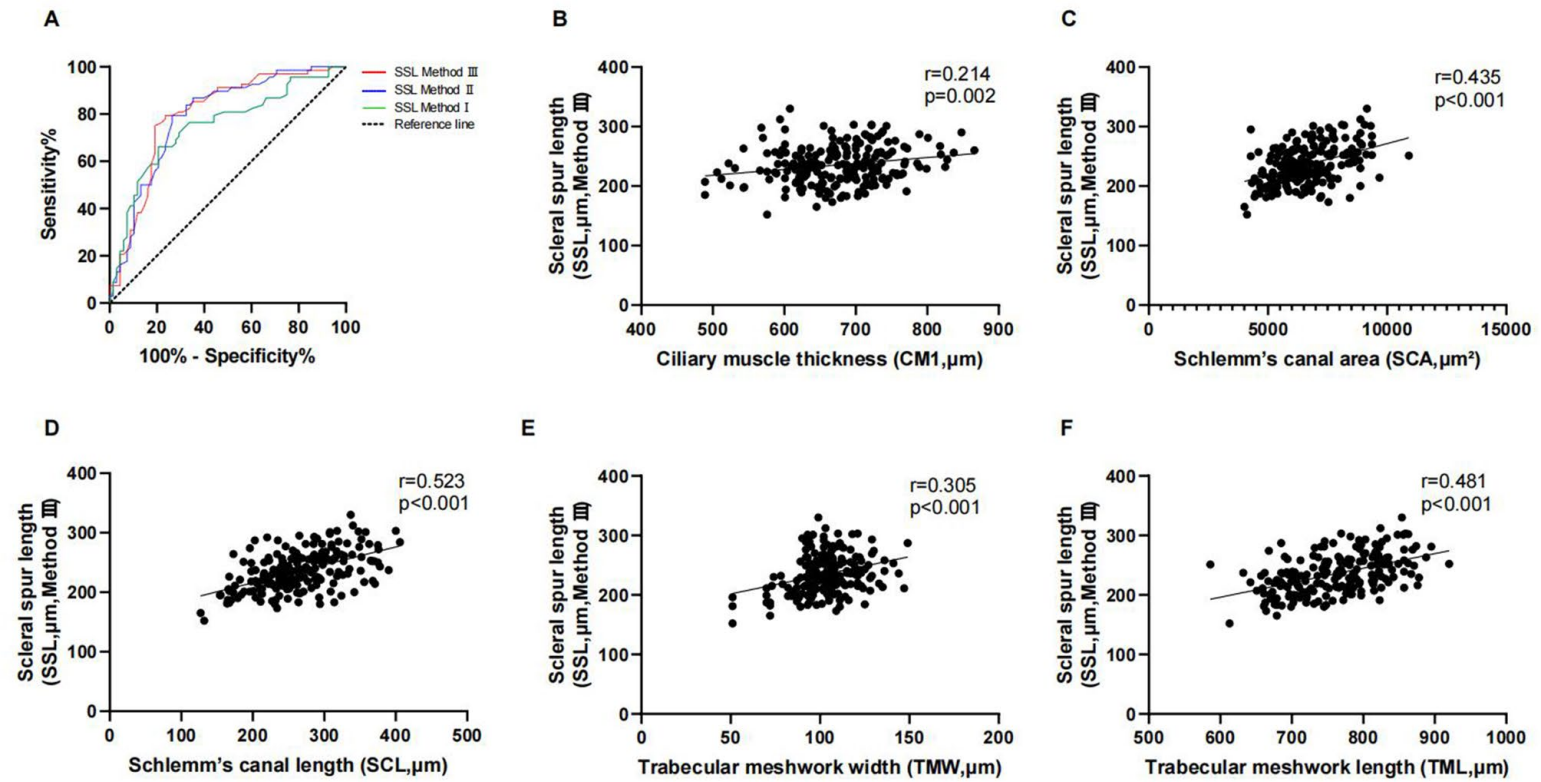
(**A**) Receiver operating characteristic curves for SSL. (**B**) CM1 was significantly correlated with SSL (r = 0.214, *p* = 0.002). (**C**) SCA was significantly correlated with SSL (r = 0.435, *p* < 0.001). (**D**) SCL was significantly correlated with SSL. (r = 0.523, *p* < 0.001). (**E**) TMW was significantly correlated with SSL. (r = 0.305, *p* < 0.001). (**F**) TML was significantly correlated with SSL (r = 0.481, *p* < 0.001).

The area under the curves (AUCs) of scleral spur length were employed to discriminate 0D from -8D and are shown in Fig. 5A. The areas under those curves for the scleral spur length were 0.749 (95% confidence interval (CI) 0.666–0.833) (Method I), 0.791 (95%CI 0.714–0.868) (Method II), and 0.798 (95%CI 0.721–0.875) (Method III), respectively. Thus, we chose the SSL measurement of Method III, which had the largest AUC, to investigate the associations of scleral spur length with chamber parameters (Fig. 5B–F). The result shows that SSL was significantly correlated with CM1, SC, and TM.

## Discussion

Ciliary muscle changes provide direct evidence of accommodation stimulation. Past research has reported that the ciliary muscle changes with accommodation using UBM, and OCT^26,27^. The ciliary muscle showed a contractile shortening and a thickening of the anterior portion with accommodation^27^. In this study, the CM1 increased significantly with accommodation state and CM3 decreased with accommodation state, consistent with past results.

In addition, we observed that the SSL increased with increasing accommodation force. We speculate that the changes in the SSL under different accommodation states may be due to several causes. On the one hand, the SS is a wedge structure formed by the projection of the inner sclera, the posterior border of the corneoscleral portion of the TM and the anterior border of the longitudinal fibers of the ciliary muscle. The ciliary muscle was thicker and showed a greater contractile response on the anterior portion^28^. Thus, the force of accommodation derived from the ciliary muscle mainly acts on the base of the SS rather than simply pulling on the tip of the SS. Moreover, elastic fiber tendons from the longitudinal fibers of the ciliary muscle are continuous anteriorly into the SS and attach to the posterior elastic fibers of the SS^8^. The wedge-shaped structure results in more longitudinal fibers of the ciliary muscle attached to the base of the SS, which results in more accommodation force derived from the ciliary muscles. On the other hand, the SS may possess contractile ability and compliance. The SS contains circumferentially oriented collagenous and elastic fibers that provide rigidity. Within the aggregated fibers of the SS, a population of circularly oriented and spindle-shaped cells (scleral spur cells) stain intensely for α-smooth muscle actin and smooth muscle myosin^29^. In addition, scleral spur cells are innervated by nerve endings, which contain granular and agranular vesicles that are regarded as typical for adrenergic terminals^30^. This evidence indicates that, in the state of accommodation stimulation, the contraction force of the ciliary muscle can posteriorly and internally pull the SS, which increases the SSL.

The TM and SC are other important structures in mediating aqueous humor outflow^3,4^. In this study, the SCA and SCL increased significantly with accommodation stimulation, and these changes showed a significantly positive association with the SSL. A previous study has reported the SC size correlated with outflow facility and influenced outflow resistance^2^. The SC inner wall and the juxtacanalicular tissue (JCT), which form the majority of outflow resistance, also respond to changes in mechanical tension^31^. Although IOP or aqueous outflow could not be simultaneously monitored during the OCT scans in our study. A previous study of young adult subjects by Read SA et al. found that IOP decreases significantly after 2 min of near fixation (under 3 D accommodation) compared with accommodation relaxed^13^. In a study comparing the influences of static and repeated accommodation on the IOP, found that both of them could significantly reduce IOP. Compared with static accommodation, the repeated accommodation did not induce a statistically significant IOP drop^14^. Stokkermans TJ et al. also reported that alternating accommodation significantly decreased IOP and a greater accommodative workload and/or longer test period may improve the effect^32^. All the results suggest that IOP may decrease with accommodation. In addition, the TML increased significantly with accommodation stimulation. We speculate that the contractile force of the ciliary muscle with the accommodation stimulation is transmitted to the TM via pulling SS, causing the TM to be stretched, which increases the TML, thus leading to an expansion of the spaces of SC. Clinically, pilocarpine is one treatment for lowering IOP in POAG, and it is a drug that increases aqueous outflow by inducing contraction of the ciliary muscle, leading to an expansion of the spaces between the beams of the TM^33^. Furthermore, the posterior part of the SC where the SS exerts the most force is wider than the anterior part, consistent with the function of the SS in maintaining the opening of the SC^24^. This evidence suggests that the SS can regulate the morphology of the TM and SC.

Most researchers suggest that accommodation stimulation could promote aqueous humour outflow and decreases IOP. However, B Kudsieh et al. used SS-OCT measured the CM length, area, and thickness. They found that CM dimensions did not differ between healthy individuals and POAG subjects^34^, this may indicate the important role of the SS in regulating the SC and TM size. In addition, a previous histological sections study by Swain DL et al. found that the significantly shorter SS in POAG eyes than in age-matched normal eye, which could not provide sufficient support for the SC and TM^10^. Li M et al. also found the scleral spur length was significantly shorter in POAG eyes compared with healthy eyes by SS-OCT in real-time and in vivo^11^. These results suggest that the shorter SS than in normal eyes contains fewer ciliary muscle fibers and TM attachments, circumferentially oriented collagenous and elastic fibers, and circularly oriented and spindle-shaped cells. The shorter SS may compromise its contractile ability and compliance. When the ciliary muscle contracts and pulls the SS of POAG eyes, it moves only a short distance posteriorly, opening few layers of meshwork beams and failing to support the SC lumen; however, this hypothesis needs further confirmation. Additionally, high myopia is an independent risk factor for open-angle glaucoma, but the reason why myopic eyes appear to be more susceptible to glaucomatous damage is unclear^35^. High myopia is characterized by a marked thinning of the sclera, choroid and retina as well as elongation of the axial length^36^. High myopia patients have a series of collagen fiber changes, including a predominantly laminar collagen fiber bundle arrangement, loss of fiber cross-links, and reductions in collagen and glycosaminoglycan synthesis^37^. Whether these changes will affect the collagen fiber structure, biochemistry or biomechanical properties of the SS in high myopia, resulting in changes in the contractile ability and compliance of SS, needs further investigation. Thus, further revealing the effect of SS contractile ability on the SC and TM may help to elucidate the underlying pathophysiological mechanisms involved in open-angle glaucoma.

There were several limitations in this study. First, we only observed SSL, SC, and TM changes in mild and moderate myopic subjects. It is unclear whether similar changes would be observed in high myopic subjects or POAG patients; thus, further verification is needed. Second, in a previous study, it was found that age had an impact on structural compliance. However, in this study, the subjects’ ages ranged from 7 to 14 years (10.91 ± 1.99 years), so whether smaller changes would occur in adults and older people requires further confirmation. Third, in the current study, when provided with accommodation stimulation, IOP or aqueous outflow could not be simultaneously monitored during the OCT scans. Future studies should employ efficient methods to evaluate outflow function may provide more insight into the mechanisms underlying the pathology of glaucoma. Last, due to the limitations of the instrument and measurement method, we only assessed the nasal quadrant.

In conclusion, using SS-OCT, we found that the SSL increased with different accommodation stimulation, and SSL was significantly correlated with SC size. These findings suggest that the contractile ability and compliance of the scleral spur play an important role in maintaining the morphology of the SC. Moreover, the force of accommodation regulates the SC size by changing the length of SS.

## Data Availability

The data sets used and analyzed for the present study are available from the corresponding authors upon reasonable request.
